# Application of 14-MHz Ultrasonography with Tissue Harmonic Imaging to Determine Posterior Capsule Integrity in Traumatic Cataract

**DOI:** 10.1155/2019/4903703

**Published:** 2019-11-12

**Authors:** Bin Wu, Qingyu Li, Yangchen Liu, Di Wu, Jianmin Gao, Yan Li, Xiaoyong Yuan

**Affiliations:** Clinical College of Ophthalmology, Tianjin Medical University, Tianjin Eye Hospital, Tianjin Key Laboratory of Ophthalmology and Visual Science, Tianjin Eye Institute, Tianjin, China

## Abstract

**Purpose:**

To report the application of 14-MHz ultrasonography with tissue harmonic imaging (14 MHz + THI) to determine the integrity of the posterior capsule (PC) in traumatic cataract (TC).

**Methods:**

Patients with TC who were scheduled to undergo cataract extraction and whose PC could not be observed by slit lamp examination were included in the study. The status of the PC was determined by 14 MHz + THI before cataract extraction and confirmed during surgery. The results regarding PC integrity obtained from 14 MHz + THI and intraoperative direct observation were compared.

**Result:**

The study enrolled 52 eyes of 52 patients (49 men and 3 women), with a mean age of 42.15 years ± 11.23 (SD). The nature of the trauma was blunt (3 eyes) or sharp (49 eyes). The 14 MHz + THI method showed 21 PCs to be intact and 31 to have ruptured before cataract surgery. During surgery, 23 PCs were observed to be intact, while 29 PCs were ruptured. 27 PCs were ruptured and 19 were intact, as determined by the two methods. The 14 MHz + THI observations were consistent with the intraoperative observations of the PC (kappa = 0.764), with no significant difference between the two methods (*P*=0.687). The sensitivity, specificity, and accuracy of 14 MHz + THI for observation of the PC were 93.10%, 82.60%, and 88.46%, respectively.

**Conclusion:**

The 14 MHz + THI method can accurately reveal the integrity of the PC in TC. It has important clinical value in the selection of cataract surgery methods and the prediction of complications during TC surgery.

## 1. Introduction

Traumatic cataract (TC) is the most common complication of ocular trauma. It is caused by direct lens injury, eye contusion, or lens dislocation. It is often accompanied by injury to the cornea, iris, and vitreum. Cataract extraction is the most effective treatment [1]. Preoperative assessment of the integrity of the posterior capsule (PC) is helpful in determining the surgical method and making more adequate preoperative preparation, avoiding improper operative techniques, and reducing the incidence of intraoperative and postoperative complications. However, after trauma, it is difficult to observe the PC by slit lamp microscopy due to corneal laceration, anterior chamber haemorrhage, lens cortical oedema and overflow, and fibrinogenesis [2].

Ultrasound and optical coherence methods are used to evaluate the state of the lens preoperatively. However, it is difficult to observe the entire lens in some cases of TC with high opacity by optical methods. Although the resolution of ultrasound is lower than that of optics, it is not affected by refractive media and it can be used to evaluate the state of the entire lens, thus meeting the needs of clinical operations [2–4]. Ultrasonography at 14 MHz with tissue harmonic imaging (14 MHz + THI) can display the whole lens and PC and is not affected by refractive medium opacity. The purpose of this study was to evaluate the clinical value of 14 MHz + THI in the examination of PC integrity in patients with TC before surgery.

## 2. Subjects and Methods

### 2.1. Subjects

This study was approved by the ethics committee of the Tianjin Eye Hospital, and all procedures were performed in accordance with the Declaration of Helsinki. Fifty-two eyes of 52 patients with TC, 49 males and 3 females, aged 17–69 years, with an average age of 42.15 ± 11.23 (range, 17–69 years), were enrolled from January 2017 to March 2018. The trauma was either sharp (47 eyes) or blunt (5 eyes), resulting in open globe injury in 49 cases and closed globe injury in the other 3 cases. The inclusion criteron was as follows: slit lamp examination could not reveal the state of the PC. The exclusion criteria were as follows: endophthalmitis, lens subluxation, and massive vitreous haemorrhage caused by retinal or choroidal injury.

### 2.2. Methods

#### 2.2.1. Preoperative Examination

All patients underwent slit lamp microscopy, 10-MHz B-scan ultrasonography, ultrasound biomicroscopy (UBM), and computed tomography (CT) examination preoperatively.

One day before the surgery, color Doppler ultrasound instrument (LOGIQ 7, GE) with a 12L probe (14 MHz, linear array scanning) at a focal point of 1-2 cm and a depth of 3 cm and THI were performed by the same experienced technician in the same indoor-brightness environment. After the patient assumed a supine position with the eyes closed, the eyelids were smeared with an appropriate amount of coupling agent; the probe should not press the eyelid directly, but should be hatched on the eyelid gently with enough coupling agent and the lens was scanned by two-dimensional ultrasound in multiple directions. If necessary, the patient was asked to rotate the eyeball to facilitate the scanning. Usually, 6–8 ultrasound sections were saved for each eye.

#### 2.2.2. Evaluation of the PC by 14 MHz + THI

The PC was evaluated in reference to a previously reported method [2]. (1) If the regular, continuous, and elliptical echoes of the posterior equatorial lens could be clearly observed, the PC of the lens was considered intact. (2) If there was a hyperechoic passage through the lens to the vitreous body, the PC was considered ruptured. (3) If there was no hyperechoic passage but the lens had lost its original elliptical shape and the PC appeared wavy and discontinuous, the PC was considered ruptured.

#### 2.2.3. Observation of the PC Intraoperatively

The PC was observed by the same two doctors during surgery. (1) If the lens showed localized cortical opacity, the opaque cortex and PC were separated using a viscoelastic agent. After removing the opaque cortex, the state of the PC was confirmed. (2) If the whole cortex was turbid and the cortex fell into the vitreous cavity, or the vitreous body overflowed when the cortex was sucked out, rupture of the PC was determined. After the cortex was removed, damage to the PC was observed. The integrity of the PC was determined by combining the above two steps.

#### 2.2.4. Statistical Methods

SPSS 17.0 statistical software was used for the statistical analysis. Two methods were applied in the same subject groups, and the results of the surgical diagnosis were considered the gold standard. The consistency of the two methods was analysed by the kappa consistency test. Kappa values <0.40, 0.40 to 0.75, and >0.75 were considered to indicate low, medium, and high consistency. The McNemar *χ*^2^ test was used to compare the distributions of the PC integrity determined by the two methods. *P* < 0.05 was statistically significant. The sensitivity, specificity, and accuracy of 14 MHz + THI were calculated. The sensitivity was calculated as true positive/(true positive + false negative). The specificity was calculated as true negative/(true negative + false positive). The accuracy was calculated as (true positive + true negative)/total number of cases.

## 3. Results

**Figure 1:**
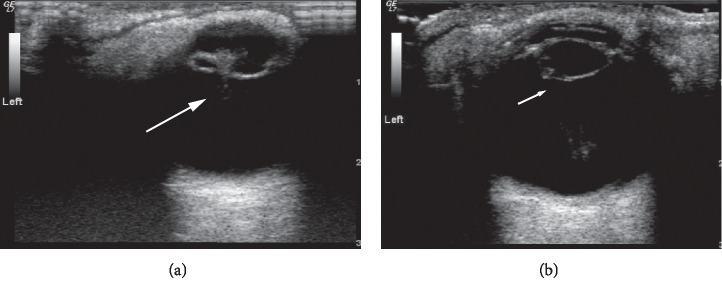
Ruptured PC shown by 14 MHz + THI. (a) A hyperechoic passage is present through the lens to the vitreous body, and the echo of the PC is interrupted (long arrow). (b) The PC shows an irregular oval echo and an uneven echo at the rupture (short arrow).

**Figure 2:**
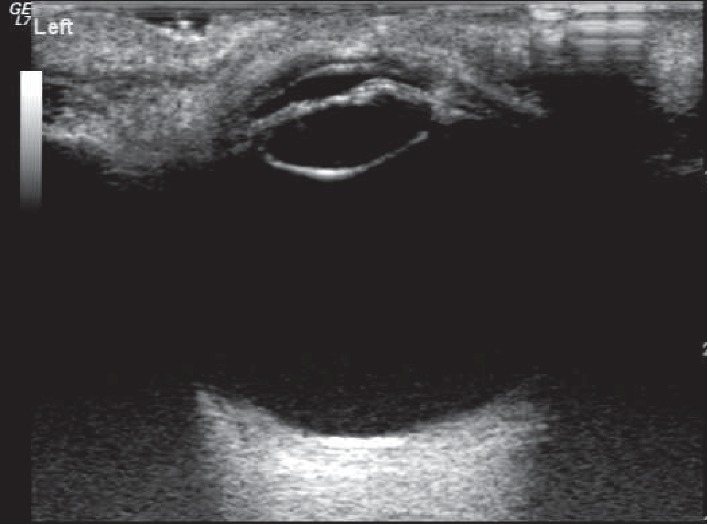
Intact PC shown by 14 MHz + THI. The shape of the lens is regular, the internal echo is uniform, the anterior capsule is ruptured, and the PC echoes are smooth and continuous.

**Figure 3:**
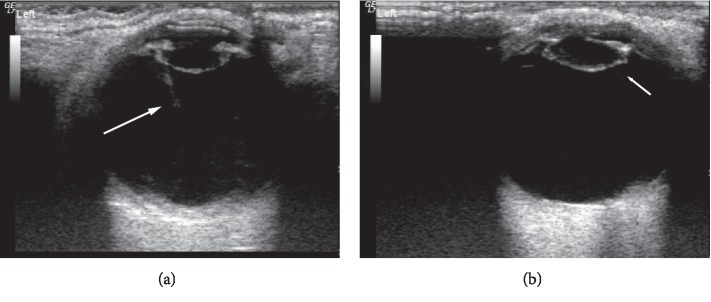
False-positive image of PC by 14 MHz + THI. (a) The shape of the lens is regular, the internal echo is uniform, and the PC echo is regular. A moderate echo band is shown and was mistaken for a passage (long arrow). (b) The shape of the lens is not regular, the internal echo is not uniform, the anterior capsule is ruptured, and the PC of the equator shows a sharp echo, which was mistaken for rupture (short arrow).

**Figure 4:**
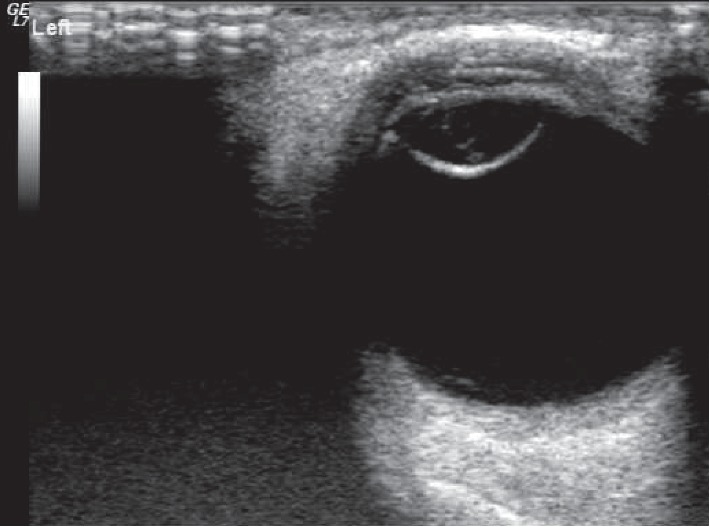
False-negative image of PC by 14 MHz + THI. The lens is dilated and regular in shape, with a cord-like medium echo and smooth, continuous PC echo.

Among the 52 eyes examined by 14 MHz + THI, PC rupture occurred in 31 eyes (Figure 1); all of these cases were of TC due to a sharp injury. A hyperechoic passage was observed through the lens to the vitreous body. Some of the lenses were seriously deformed, and the PC was discontinuous. The PC of the lens was intact in 21 eyes (Figure 2) and had sustained a sharp injury in 16 eyes and had sustained a blunt injury in 5 eyes. The posterior equatorial lens was oval in shape, with continuous echoes of the PC, and the anterior capsule of some lenses was ruptured. For 6 eyes, the experimental results were not in accordance with the results of surgery; the results of 4 eyes were false positive (Figure 3), those of 2 eyes were false negative (Figure 4), and all of these eyes had sustained a sharp injury. There was no significant difference in the PC integrity determined by 14 MHz + THI and intraoperative observation (kappa = 0.764, *P*=0.687; sensitivity = 93.10%, specificity = 82.60%, and accuracy = 88.46%).

## 4. Discussion

The integrity of the PC plays a key role in the implantation of intraocular lenses and the location of implantation. Pre- and postoperative evaluations of the integrity of the PC are necessary. In this study, color Doppler ultrasound showed the advantages of adjustable focus and detection depth compared with B-scan ultrasound. THI is a routine component of ultrasound (US) diagnosis , in which, higher-frequency harmonic waves produced by nonlinear fundamental ultrasonography wave propagation are used to generate images that contain fewer artifacts than those seen on conventional fundamental wave US tissue imaging. The advantages of THI include the following: improved contrast resolution, improved lateral resolution and reduced section thickness, beneficial effects on artifacts, reduced noise in the near field, and improved imaging of deeper tissue [5].

The 14 MHz + THI method can be used to not only display images of the whole eyeball, but also observe the whole lens and PC. In this study, there was no significant difference in PC integrity as determined by 14 MHz + THI and intraoperative observation. The accuracy of 14 MHz + THI was 88.46%, indicating that it can be used to evaluate PC integrity before surgery. In this study, there were 2 false-negative results for eyes with a moderate echo passage in the lens: a smooth and continuous PC echo. No cortical spillover or vitreous opacity was observed around the lens. It was considered that the passage is not penetrating the PC. This study suggests that in TC, small and shallow ruptures can close quickly after trauma, resulting in localized opacity, or that the PC and cortex of the wound can be close to each other. In such cases, it is difficult to observe rupture of the PC. In addition, TC patients may also experience rupture or relaxation of the ciliary zonule. Iatrogenic capsule rupture easily occurs when the cortex is removed. This is not excluded from occurring in patients with false-negative findings [6]. In 4 eyes with false-positive lenses, the anterior capsule of 3 eyes had a larger fissure, more cortex spilled into the anterior chamber, the PC tension decreased, the lens lost its original oval echo, and the PC echo was wavy, all of which contributed to the mistaken identification of rupture. In one eye, the vitreum around the equatorial PC showed a strip-like echo, which was mistaken as a passage through the lens to the vitreum, indicating PC rupture. In cases of more serious ocular penetration injury, the cortex overflows more, the lens deforms, and either the PC loses its smooth regular echo or a strip-like echo is observed around the equatorial PC, which can be mistaken as a penetration passage; these two situations are liable to lead to the misdiagnosis of PC rupture.

There are many methods for observing the PC of the lens preoperatively. The shorter the ultrasound wavelength, the higher the image resolution, and the shallower the tissue penetration [7]. UBM at frequencies up to 50–100 MHz allows clear visualization of the anterior segment of the eye, but the shorter wavelengths limit the depth of detection (<5 mm) and prevent observation of the whole lens and PC [8, 9]. When the frequency of UBM is decreased, the detection depth increases, and the PC of the lens can be observed. Kucukevcilioglu et al. [10] applied 35-MHz UBM to clearly identify a PC wound less than 1 mm in size. Because of its good penetration and high resolution, high-frequency B-scan ultrasonography avoids the near-field interference of ultrasonography and is used to observe the PC in TC [2, 4, 11]. Tabatabaei et al. [2] examined 43 TC eyes with 20-MHz high-frequency B-scan ultrasonography before and after surgery. The accuracy was 88.37%, which was similar to that observed in this study. However, both methods require surface anaesthesia and immersion measurements, which not only increase the risk of corneal injury when the wound is not completely closed, but also increase the risk of intraocular infection. At the same time, a degree of patient cooperation and surgical skill are required. The 14 MHz + THI method does not require surface anaesthesia or immersion and can be performed regardless of whether the wound is closed, thus avoiding the risks of corneal injury and intraocular infection. This method is simple to perform, and both children and elderly patients cooperate easily. Anterior segment optical coherence tomography (AS-OCT) and Scheimpflug imaging can be used to observe and analyse the anterior segment structure clearly and objectively, scan the lens comprehensively, measure the lens thickness, and evaluate the PC [12–17]. Yang et al. [18] used Pentacam to diagnose foreign bodies in lenses, locate foreign bodies accurately, and detect the integrity of the PC of the lens. Recently, it has been reported that using the three-dimensional (3D) visualization with the long-range swept source optical coherence tomography (SS-OCT) system, the subtle opacities of the cataractous crystalline lens could be characterized quantitatively and located accurately, which might be a more useful tool for the observation on the details of the PC of TC [19, 20]. Based on the principles of optical imaging, this method has the advantages of high resolution, no contact, simple operation, and no risk of infection. However, when the lens opacity is severe or the lens expansion exceeds the detection range, the above methods cannot be used to evaluate or observe the PC effectively.

However, the sample size of our study was limited, especially for the blunt trauma cases, in which all the PCs were intact. With more cases of blunt trauma, the feature of PC ruptures detected by 14 MHz + THI could be added.

## 5. Conclusion

In this study, we found that 14 MHz + THI in cases of TC are simple and risk-free. This method can be used to clearly observe the PC preoperatively and thus guide the operation and reduce the risk of surgery.

## Data Availability

The data used to support the findings of this study are available from the corresponding author upon request.
